# Extending the Omega model with momentum and reversal strategies to intraday trading

**DOI:** 10.1371/journal.pone.0291119

**Published:** 2023-09-08

**Authors:** Jing-Rung Yu, Chieh-Hui Wei, Chi-Ju Lai, Wen-Yi Lee

**Affiliations:** 1 Department of Information Management, National Chi Nan University, Nantou, Taiwan; 2 Department of Information Management, National Taipei University of Business, Taipei, Taiwan; University of Durham: Durham University, UNITED KINGDOM

## Abstract

This study develops the Omega model integrated with momentum and reversal strategies using high-frequency data on the component stocks of the S&P 500 Index and the NASDAQ 100. The Omega model based on the momentum strategy (M_Omega), the reversal strategy (R_Omega), and both strategies (M_R_Omega) are designed to simulate trading over three periods. The portfolio is rebalanced every transaction day to optimize asset allocation by incorporating intraday winners or losers’ information and trading cost. The study finds that the proposed models generate positive returns (net of trading costs), in spite of fact that intraday trading frequently erodes profits. The M_Omega and R_Omega models produce a higher return than that of the S&P 500 index or NASDAQ 100 index, considering the intraday trading cost. The performance of the Omega model integrated with the momentum or reversal strategy is more profitable in a volatile market or period. The M_Omega and R_Omega reach the highest final market value from 2020 to 2021, when COVID 19 pandemic emerged. The rebalancing of the momentum or reversal strategy is suitable for the short term but not recommended in the long term for intraday trading as the trading costs become increasingly significant over time.

## 1. Introduction

The reversal and momentum effects on security returns has been an important topic for investment. De Bondt and Thaler [1] have shown that stock prices overreact to news, suggesting that contrarian or reversal strategies, buying past losers and selling past winners, achieve abnormally high returns. They indicate that, over 3- to 5-year holding periods, stocks that perform poorly over the previous 3- to 5-years have higher returns than stocks that perform well over the same period. De Bondt and Thaler’s [1] results are consistent with the overreaction hypothesis. The overreaction effect is asymmetric: it is much more significant for losers than for winners. Portfolios of prior “losers” are found to outperform prior “winners.” Thirty-six months after portfolio formation, the losing stocks have earned about 25% more than the winners, despite the latter being significantly riskier [1]. Parhizgari and Nguyen [2] have shown the profitability of contrarian strategies in the U.S. stock market employing American Depository Receipts (ADR). The contrarian strategy is also profitable in the Chinese stock market [3]. Li [4] finds that the lower a stock gets, the greater the potential reversal effect while dramatic change of the investor sentiment being above the upper critical value. Kim and Suh [5] also observe short term reversals in returns.

On the other hand, Jegadeesh [6] shows evidence of return continuation and momentum in stock returns: assets that perform well in the past will perform well in the future. Jegadeesh and Titman [7] argue that, if stock prices either overreact or underreact to information, profitable trading strategies that select stocks based on their past returns exist. They find that buying past winners and selling past losers, forming a long-short portfolio of those stocks, generates statistically significant and economically relevant returns throughout a holding period of one year. See Fama [8] for broad evidence on return continuation, who calls momentum the premier unexplained financial anomaly. Griffin et al. [9] have shown that this type of momentum is expected in global stock markets. In addition to this cross-sectional momentum, Moskowitz et al. [10], Asness et al. [11], and Huang et al. [12] examine whether there is an annual time-series momentum. They find that the previous 12-month returns of an asset positively predict its future returns. Consistent with Jegadeesh and Titman [13], Zhu et al. [14] find that the simple momentum strategy generates significant and positive returns in the subsequent year but insignificant or even significant negative returns thereafter. Elaut et al. [15] suggest that market concentration due to trading hours matters for intraday momentum and that the effect is more pronounced during financial crises.

Some researchers document the relationship between momentum and reversal strategies. Specifically, Conrad and Kaul [16] argue that contrarian strategies are profitable for short-term (weekly, monthly) and long-term (3- to 5-years, or longer) intervals, while the momentum strategy is profitable for medium-term (3- to 12-month) holding periods. Jegadeesh and Titman [13] have emphasized that momentum and reversal phenomena are intimately linked. Over a longer time horizon, they find that return reversal often follows return continuation, consist with findings of Hofmann et al. [17].

While most forms of momentum or reversal strategies are examined at monthly, weekly, or daily frequencies, the rise of technology has led to a substantial increase in high-frequency trading (HFT) research. And as the high-frequency data become more available, researchers are examining the momentum and reversal strategies in terms of intraday trading. As noted by Malceniece et al. [18], the scale of HFT activity varies depending on the market and how broadly HFT is defined, but there is no doubt that HFT accounts for a large share of trading volume in most developed markets. The availability of HFT has changed how traders trade, how markets are structured, and how liquidity and price discovery arise [19]. HFT has thus had a fundamental impact on markets, propelling many academics to examine trading behavior in financial markets at a much higher frequency [20, 21].

Based on high-frequency data for the S&P 500 ETF from 1993 to 2013, Gao et al. [22] have shown that an intraday momentum pattern occurs during the first half-hour return on the market as measured from the previous day’s market close, which positively explains the last half-hour return. This predictability is significant in the sample and out of the sample. Similar to Gao et al. [22], Onishchenko et al. [23] suggest the first half-hour return as the trading signal based on equally weighted asset allocation yields the highest portfolio return. One necessary condition for implementing these strategies is that all positions must be closed at the market close on each trading day and re-opened on the following day. Li et al. [24] conduct a cross-country study on intraday momentum based on the work of Gao et al. [22]. They provide strong evidence of intraday time series momentum that the first half-hour return of the trading day significantly predicts the last half-hour return in a selection of U.S. exchange-traded funds (ETFs).

Herberger et al. [25] extend the intraday reversal and momentum strategies explored in De Bondt and Thaler [1] and Jegadeesh and Titman [7] by shortening the time horizon to intraday trading. To do so, 16 reversal and 16 momentum strategies with ranking periods and holding periods of 60, 120, 180, or 300 minutes in the case of reversal strategies and 15, 30, 45, or 60 minutes in the case of momentum strategies among German blue chip stocks traded on XETRA are analyzed. The descriptive results indicate that intraday reversal strategies can generate statistically significant positive returns but with high risk. All strategies generate significant positive market-adjusted returns, meaning that the price of the stock bought due to the reversal strategy rose more than the market proxy, especially for the reversal strategy ranking 180 minutes to pick up losers and holding 60 minutes perform best. Nevertheless, Herberger et al. [25] point out that the strategies’ returns are insufficient to cover the transaction fees XETRA charges.

Therefore, how to employ the reversal or momentum strategy using high frequency data to obtain the positive return after taking into account trading cost is essential in intraday trading. Moorman [26] notes that momentum strategies involve frequent rebalancing based on changes in the returns of individual stocks, implying a significant difference in performance before and after trading costs. In previous studies, for simplicity, researchers primarily use the equally-weighted asset allocation to evaluate portfolio performance, ignoring trading cost [8–10, 12, 14, 25, 27, 28].

In contrast to an equally-weighted asset allocation, the Omega model [29] uses a given return threshold to gauge gain and loss, generating optimal asset allocation weights by maximizing the deviation of the gain to loss. For intraday trading, the investor tends to be risk seeking. In order to define the gain and loss, a given return threshold in the Omega model can meet the requirement of the risk seeking investor. Instead of using an equally weighted asset allocation, we integrate the Omega model [29], a linear model taking into gain and loss for large-scale asset allocation with momentum and reversal strategies under consideration of the trading cost while rebalancing [30]. For focusing on the effect of the momentum or reversal strategy, we omit the short selling and consider two objectives: maximizing the deviation between portfolio gain and loss, and minimizing the transaction cost.

We examine the momentum, reversal, and momentum-reversal strategies and follow Onishchenko et al. [23], Herberger et al. [25] and Hofmann et al. [17] by using thirty minutes as an interval for high-frequency data collection. For the momentum strategy, we select winners by ranking the first half-hour return of all assets. For the reversal strategy, we select losers by ranking the first six half-hour returns of all assets. For the momentum-reversal strategy, the winners are purchased at 10:00 am and the loser stocks are purchased at 12:30 pm. These three strategies combine the Omega model [29], M_Omega, R_Omega, and M_R_Omega, to allocate assets rather than use an equally-weighted approach for the winners and losers. We estimate the model performance using the composite stocks of the S&P 500 and the NASDAQ 100 for three periods: 1/1/2018–4/22/2019, 1/1/2020–4/22/2021, and 1/1/2021–4/22/2022. For daily rebalancing, the market values of M_Omega, R_Omega, and M_R_Omega are calculated at market close (4:00 pm).

This study has four contributions. First, limiting the maximum number of assets for daily rebalancing avoids over-diversification. Second, the proposed models generate positive returns and overcome the drawback that intraday trading frequently erodes profits, resulting in negative returns [25]. Third, considering intraday trading cost, the Omega model integrated with the momentum or reversal strategy performs better than the S&P 500 or NASDAQ 100 index. Finally, a combined strategy, performing the momentum and reversal strategies on the same day, is not recommended as the trading cost grows dramatically over time.

The rest of the paper is organized as follows: Section 2 introduces the Omega model with momentum and/or reversal strategies; Section 3 shows the experimental results; Section 4 concludes.

## 2. The Omega model without/with trading strategies

This study uses four types of models: Omega, M_Omega, R_Omega, and M_R_Omega, with a daily rebalancing mechanism considering trading cost [30]. The Omega model serves as the benchmark. The M_Omega model buys winners for intraday trading, the R_Omega model buys losers for intraday trading, and the M_R_Omega model buys winners and losers for intraday trading.

### 2.1 The rebalancing Omega model

Keating and Shadwick [31] develop the Omega ratio, the percentage of the gain over loss given the return threshold, *τ*, by the investor as

$$\omega =\frac{E\text{(}r_{j})-\tau }{E{\text{[}\tau -r_{j}]}^{+}}+1,$$

where *r*_*j*_ is the return for the *j*^th^ asset; *E*(*r*_*j*_) is the expected return for the *j*^th^ asset; *E*[*τ* − *r*_*j*_]^+^ is the expected loss below the return threshold. Kapsos et al. [29] proposed a worst-case Omega model, a linear portfolio model that handles uncertainty in asset return under three scenarios. When the number of scenarios equals one, the model is reduced to a simple Omega model. Yu et al. [30] extend this simple Omega model considering trading cost for portfolio rebalancing. Their model considers three objectives: maximizing the deviation between portfolio gain and loss, minimizing the short selling proportion, and minimizing the transaction cost. To focus on the momentum and reversal strategies, we omit short selling and consider two objectives: maximizing the deviation between portfolio gain and loss, and minimizing the transaction cost. Trading cost is minimized for each rebalancing to avoid overwhelming trading assets. Further, we used the simple weighted method to reformulate the multiple-objective model into one objective model in Eq (1).

**Omega model**:

$$\text{Max}\;\psi -\sum_{j=\text{1}}^{n}\text{(}c_{\text{1}}l_{j}^{+}+c_{\text{2}}l_{j}^{-}\text{)}$$ (1)

$$\text{s.t.}\;\delta (\sum_{j=1}^{n}\text{(}w_{j}{\bar{r}}_{j}-\tau )-(1-\delta )\frac{1}{T}\sum_{t=1}^{T}\eta_{t}\geq \psi ,$$ (2)

$$\eta_{t}\geq -\sum_{j=1}^{n}r_{j,t}w_{j}+\tau ,\;\text{for}\;t=\text{1},\text{2,}\ldots \text{,}T,$$ (3)

$$\eta_{t}\geq \text{0},\;\text{for}\;\;t=\text{1},\text{2,}\ldots \text{,}T,$$ (4)

$$\sum_{j=\text{1}}^{n}\text{(}w_{j}+c_{\text{1}}l_{j}^{+}+c_{\text{2}}l_{j}^{-}\text{)}=\text{1,}$$ (5)

$$w_{j}=w_{j,0}+l_{j}^{+}-l_{j}^{-},\;\text{for}\;j=\text{1},\text{2,}\ldots \text{,}n,$$ (6)

$$0.01u_{j}\leq w_{j}\leq u_{j}\text{,}\;\text{for}\;j=\text{1},\text{2,}\ldots \text{,}n,$$ (7)

$$u_{j}\in \left\{0,1\right\}\text{,}\;\text{for}\;j=\text{1},\text{2,}\ldots \text{,}n,$$ (8)

$$w_{j},w_{j}^{\text{0}}\text{,}l_{j}^{+}\text{,}l_{j}^{-}\geq \text{0,}\;\text{for}\;j=\text{1},\text{2,}\ldots \text{,}n,$$ (9)

where *τ* is the return threshold specified by the investor, ${\bar{r}}_{j}$ and *r*_*j*,*t*_ are the average return and the return of the *j*^*th*^ asset on the *t*^*th*^ day, respectively and *w*_*j*_ is the weight of asset *j* invested after portfolio rebalancing. Eqs (3) and (4) measure the loss of the portfolio, *η*_*t*_, on the *t*^*th*^ day. Eq (5) is the budget constraint reflecting various transaction costs. At the same time, in rebalancing, *c*_*j*_ (*j* = 1, 2) represents the transaction costs of buying and selling the *j*^*th*^ asset, respectively. Since it is a self-financing portfolio, the budget for long positions and trading costs is normalized to 1. Eq (6) shows the weight change before and after rebalancing. *w*_*j*,0_ refers to the long position of the *j*^*th*^ asset of the portfolio market value before portfolio rebalancing; $l_{j}^{+}$ and $l_{j}^{-}$ represent the weights for buying and selling the *j*^*th*^ asset. The upper (100%) and lower (1%) bounds for the long and short positions for each security are included in the constraints (Eqs (7) and (8)). The binary variables *u*_*j*_ are used to identify the long positions.

Notably, *u*_*j*_ are decision variables in the rebalancing of the Omega model. We can specify some of the *u*_*j*_ to be 1 to ensure a long position in the *j*^*th*^ asset. We can therefore integrate winners or losers from the momentum or reversal strategy into the Omega model. Then the Omega model optimizes the allocation weights for winners, losers, and the other securities.

### 2.2 The Omega with momentum/reversal strategy

We extend the above Omega model to allow for long positions in the momentum and reversal strategies (M_Omega, R_Omega and M_R_Omega) while doing intraday rebalancing. Herberger et al. [25] use the composite stocks of DJX, which include 30 stocks, and identify top ten percent of stocks and bottom ten percent as winners and losers, respectively. Since we apply the model to the broader composite stocks of the S&P500 and NASDAQ100, we select only the top or bottom one (or two) percent of stocks as winners or losers for each trading day. Consider the composite stocks of the S&P 500 as an example. For the momentum strategy, after ranking the first half-hour returns, $r_{\text{1},\;t}=\frac{p_{\text{10:00},\;t}}{p_{\text{9:30},\;t}}-1$, the five highest-return stocks are chosen as winners and the five lowest-return stocks are chosen as losers, where *p*_9:30,*t*_ and *p*_10:00,*t*_ represents the stock’s price at 9:30 and 10:00 am on day *t*, respectively. Gao et al. [22], Onishchenko et al. [23], and Li et al. [24] have shown a positive return using the first half-hour return to predict the last half-hour return in the intraday momentum strategy. Following their framework, we use the first half-hour return for the intraday momentum strategy. The M_Omega model employs the momentum strategy and forces the purchase of winners as follows.

**M_Omega**:

$$\text{Max}\;\psi -\sum_{j=\text{1}}^{n}\text{(}c_{\text{1}}l_{j}^{+}+c_{\text{2}}l_{j}^{-}\text{)}$$

$$\text{s.t.}\;\delta \text{(}\sum_{j=1}^{n}w_{j}{\bar{r}}_{j}-\tau \text{)}-(1-\delta )\frac{1}{T}\sum_{t=1}^{T}\eta_{t}\geq \psi ,$$

$$\eta_{t}\geq -\sum_{j=1}^{n}r_{j,t}w_{j}+\tau ,\;\text{for}\;t=1,2,\ldots ,T,$$

$$\eta_{t}\geq \text{0},\;\text{for}\;t=1,2,\ldots ,T,$$

$$\sum_{j=\text{1}}^{n}\text{(}w_{j}+c_{\text{1}}l_{j}^{+}+c_{\text{2}}l_{j}^{-}\text{)}=\text{1}$$

$$w_{j}=w_{j,0}+l_{j}^{+}-l_{j}^{-},\;\text{for}\;j=1,2,\ldots ,n,$$

$$0.01u_{j}\leq w_{j}\leq u_{j},\;\text{for}\;j=1,2,\ldots ,n,$$

$$u_{j}=1,\;\text{for}\;\;j\in L,$$ (10)

$$u_{j}\in \{0,1\},\;\text{for}\;j=1,2,\ldots ,n,\;\text{and}\;j\notin L,$$ (11)

$$w_{j},w_{j}^{\text{0}}\text{,}l_{j}^{+}\text{,}l_{j}^{-}\geq \text{0,}\;\text{for}\;j=1,2,\ldots ,n.$$

Note that Eq (10) identifies the winners which belong to the long set (*L*), “must buy” (*u*_*j*_ = 1), in the model at 10:00 am, but the allocation weights are generated from the Omega model. We do not use an equally-weighted method to decide the allocation weights for the winners or losers, as do Jegadeesh and Titman [7], Huang et al. [12], Zhu et al. [14], Onishchenko et al. [23], and Herberger et al. [25]. The M_Omega model optimizes the allocation weights, including the winners in Eq (10) ranked by the momentum strategy and other stocks in Eq (11), according to historical returns.

The details of the procedure for the M_Omega model are

1. Select winners by ranking the returns of the composite stocks at 10:00 am;
2. Assign all winners to the *L* set;
3. Let *u*_*j*_ = 1 for all stocks in the *L* set, which must be purchased under the Omega model;
4. Run the M_Omega model based on the return of the past 60 days for each stock at 10:00 am;
5. Calculate the market value of the portfolio at 4:00 pm, and
6. Rebalance the portfolio by repeating steps (1) to (5) for every transaction day.

**R_Omega**:

Herberger et al. [25] have shown that the best reversal timing is to rank for the first six half-hour returns after the market opens. We follow their reversal strategy and buy the losers at 12:30 pm. The R_Omega model requires buying the losers according to the reversal strategy and the corresponding *u*_*j*_ being assigned one (Eq (10)); the rest of the chosen stocks are generated by the Omega model (Eq (11)). The allocation weights for the losers and for the rest of the chosen stocks are generated by the Omega model. The difference between the M_Omega and R_Omega models is in Eqs (10) and (11): the winners belong to *L* set for the M_Omega whereas the losers belong to the *L* set for the R_Omega model.

The details of the procedure for the R_Omega model are

1. Select losers by ranking the returns of the composite stocks at 12:30 pm;
2. Assign all losers to the *L* set;
3. Let all *u*_*j*_ = 1 in the *L* set, which must be purchased under the Omega model;
4. Run the R_Omega model based on the return of past 60 days for each stock at 12:30 pm;
5. Calculate the market value of the portfolio at 4:00 pm, and
6. Rebalance the portfolios by repeating steps (1) to (5) for every transaction day.

**M_R_Omega**:

For a hybrid trading strategy, we combine the momentum and reversal strategies in intraday trading, where the M_R_Omega model allows purchasing winners at 10:00 am and then purchasing losers at 12:30 pm. That is we run the M_Omega model at 10:00 to generate the allocation weights of the winners and the other stocks first and then run the R_Omega model at 12:30 pm to generate the weights of the losers and the other stocks within a trading day.

## 3. Experiments and results

We apply the model to the S&P 500 and NASDAQ 100 stocks. Specifically, we use 30-minute tick data on the composite stocks of the S&P 500 and the NASDAQ 100 from 1/1/2021 to 4/22/2022, 1/1/2020 to 4/22/2021, and 1/1/2018 to 4/22/202019. Excluding missing data, sample details are depicted in Table 1. The returns of sixty days are employed to construct the initial portfolio, which is rebalanced daily to compare the intraday trading strategies. The return threshold (*τ*) is set at 0.1% and the transaction costs of buying (*c*_1_) and selling (*c*_2_) are both set at 0.025% for the Omega model. Regardless of the momentum strategy and/or reversal strategy, the market values of the portfolios are calculated when the market closes. The Omega model and market index returns including the S&P 500 and NASDAQ 100 are using as benchmarks. The optimization software used in this study is Lingo 18.0 on a computer with Intel ^®^ Core^™^ i7-10700 CPU @ 2.90GHz, NVIDIA GeForce RTX 3060, Windows 10.

**Table 1:** Summary information for the S&P and NASDAQ datasets.

Period	Composite Stocks	Trading days	Number of assets	Rebalancing times
1/1/2021 to 4/22/2022	S&P500	306	446	246
	NASDAQ 100	326	69	266
1/1/2020 to 4/22/2021	S&P500	321	444	261
	NASDAQ 100	330	69	270
1/1/2018 to 4/22/202019	S&P500	315	444	255
	NASDAQ 100	321	69	261

### 3.1 Experiment 1: Portfolios with no limits on the number of invested assets

The first experiment using the composite stocks of the S&P 500 during 1/1/2021 to 4/22/2023 involved considering only buying stocks for each of the M_Omega, R_Omega, and M_R_Omega models. As depicted in Table 2, the Omega model, limited to daily rebalancing, produces the highest daily return and highest return standard deviation (0.063% and 1.31%). The return of the S&P 500 index is 0.057% but with the lowest risk (0.95%). The Sharpe ratio of the S&P 500 index is the highest (0.060) because of the lowest standard deviation for this passive investment. The Omega model excludes buying winning or losing stocks according to momentum or reversal strategies. Therefore, 83 out of 246 rebalancing times remain the same on the allocation weight of assets as the previous day, so the average transaction cost and the turnover rate are the lowest, at $61 and 0.033, respectively.

**Table 2:** Portfolio comparisons for the of S&P 500 dataset without limiting the number of assets.

Panel A: Return						
Model	Average return (%)	Standard deviation (%)	Max return (%)	Min (%)	Sharpe Ratio	
M_Omega	0.025	0.99	2.69	-2.87	0.025	
R_Omega	0.035	1.04	2.91	-2.81	0.033	
M_R_Omega	0.011	1.00	2.82	-3.09	0.012	
Omega	0.063	1.31	3.80	-3.78	0.048	
S&P 500	0.057	0.95	2.57	-2.95	0.060	
Panel B: Market values						
Model	Final market value ($)	Average market value ($)	Average number of buying assets ($)	Average trading cost ($)	Accumulated trading costs ($)	Average turnover rate
M_Omgea	1,046,965	1,048,056	68	222	54,500	0.091
R_Omega	1,070,228	1,072,303	53	246	60,402	0.100
M_R_Omega	1,013,121	1,044,272	125	435	107,091	0.506
Omega	1,126,511	1,089,328	15	61	15,049	0.033

Now consider the results for the other models. The final market value of the R_Omega model is higher than the M_Omega model, with average returns of 0.035% and 0.025%, respectively. The final market value of the M_R_Omega is the lowest of the four models. Table 2 also shows that the over-diversified issue occurs in the M_Omega, R_Omega, and M_R_Omega models. These models have larger number of invested assets than the number generated by the Omega model. Based on the momentum or reversal strategies, the M_Omega, R_Omega, and M_R_Omega models require buying the five winners, five losers, and five winners and five losers on the same day, respectively. Therefore, the average amount of invested assets increase over time, to 68, 53, and 125 for the three models, respectively. In contrast, the average number for the Omega model is 15. Thus, rebalancing the intraday trading with momentum or reversal strategy causes over-diversified portfolios.

### 3.2 Experiment 2: Portfolios with limited number of invested assets

To cope with the over-diversified issue, we set an upper bound of 15 assets to the M_Omega, R_Omega, and M_R_Omega models during the 1/1/2021 to 4/22/2022 period. The R_Omega and Omega models produce higher returns than does the S&P 500 index. The return of the R_Omega model (0.077%) is the highest among the four models, as depicted in Table 3, with final and average market values of $1,173,843 and $1,105,545. In general, the trading cost of the Omega model is the lowest because the only trades involve rebalancing the portfolio. Regardless of the momentum or reversal strategy, Herberger et al. [25] note that the frequent transactions of intraday trading causes heavy trading costs, eroding the portfolios’ profits and even resulting in negative returns. However, as shown in Table 3, despite incurring trading cost, the return of the R_Omega model exceeds the return of the S&P 500 Index due to its intensive asset reallocation. The trading costs for the M_Omega, R_Omega, and M_R_Omega models are higher than their corresponding values in Table 2 after limiting the buying to 15 assets in the portfolios.

**Table 3:** Portfolio comparisons for the S&P 500 dataset with buying limited to 15 assets, from 1/1//2021 to 4/22/2022.

Panel A: Return						
Model	Average return (%)	Standard deviation (%)	Max return (%)	Min (%)	Sharpe Ratio	
M_Omega	0.055	1.36	3.67	-4.09	0.040	
R_Omega	0.077	1.42	3.75	-3.76	0.054	
M_R_Omega	0.022	1.38	4.59	-3.56	0.016	
Omega	0.063	1.31	3.80	-3.78	0.048	
S&P 500	0.057	0.95	2.57	-2.95	0.060	
Panel B: Market value						
Model	Final market value ($)	Average market value ($)	Average number of buying assets ($)	Average trading cost ($)	Accumulated trading costs ($)	Average turnover rate
M_Omega	1,115,054	1,084,522	15	306	75,178	0.122
R_Omega	1,173,843	1,105,545	15	308	75,693	0.120
M_R_Omega	1,028,218	1,022,259	30	528	129,875	0.511
Omega	1,126,511	1,089,328	15	61	15,049	0.033

For each daily rebalancing, the trading cost is $p_{1}l_{j}^{+}+p_{2}l_{j}^{-}$ for all portfolios with buying and selling of the *j*^th^ asset. We calculate the average turnover for each period by the average of the sum of all the absolute changes in portfolio weights for 246 rebalancing trades: $\sum_{i=\text{2}}^{246}\sum_{j=1}^{446}|w_{i,j}-w_{(i-1),j}|/\text{(}246-1\text{)}$. As shown in Table 3, the turnover rate when asset accumulation is limited to 15 assets are greater than those models without asset accumulation limits, especially for M_Omega, and R_Omega models in Table 2. Limiting asset accumulation to 15 leads to more intensive asset reallocation, which results in a surge in trading cost. The more intensive reallocation raises the market value of the portfolios as well, as opposed to a strategy of merely cutting trading cost to increase portfolio value. The reason why the trading cost increases, after limiting the number of assets to 15, is the more frequent weight changes ($l_{j}^{+},\;\text{and}\;l_{j}^{-}$) in rebalancing. More frequent weight changes occur with the Omega model with the momentum or reversal strategy. Therefore, the turnover rate of the M_R_Omega model is the highest, however, as the portfolio is rebalanced twice each trading day, resulting in the highest transaction cost and lowest final market value.

### 3.3 Experimental results from different markets with three periods

The third experiment limits the number of stocks for each rebalancing to be less than or equal to the average number in the Omega model for the three periods. We compare the results for the composite stocks of the S&P 500 and NASDAQ 100 for three periods, where five winners or losers are chosen from the S&P 500 and two winners or losers are chosen from the NASDAQ 100. Tables 3–8 depict the performances of all portfolios in the S&P 500 and NASDAQ 100 markets for three periods. The returns of the M_Omega, R_Omega and M_R_Omega are positive, net of trading costs.

**Table 4:** Portfolio comparisons for the NASDAQ 100 dataset from 1/1/2021 to 4/22/2022.

Panel A: Return						
Model	Average return (%)	Standard deviation (%)	Max return (%)	Min return (%)	Sharpe Ratio	
M_Omega	0.102	1.18	5.70	-3.13	0.086	
R_Omega	0.107	1.24	5.72	-3.58	0.086	
M_R_Omega	0.041	0.91	4.85	-3.71	0.046	
Omega	0.110	1.31	6.03	-4.52	0.084	
NASDAQ 100	0.025	1.39	4.03	-4.22	0.018	
Panel B: Market value						
Model	Final market value ($)	Average market value ($)	Average number of buying assets ($)	Average trading cost ($)	Accumulated trading costs ($)	Average turnover rate
M_Omega	1,292,828	1,189,737	9	135	36,019	0.053
R_Omega	1,299,859	1,190,409	9	142	37,711	0.054
M_R_Omega	1,224,790	1,156,716	18	117	62,326	0.074
Omega	1,274,265	1,182,436	9	42	11,049	0.023

**Table 5:** Portfolio comparisons for the S&P 500 dataset from 1/1/2020 to 4/22/2021.

Panel A: Return						
Model	Average return (%)	Standard deviation (%)	Max return (%)	Min return (%)	Sharpe Ratio	
M_Omega	0.407	2.84	8.39	-13.08	0.143	
R_Omega	0.335	2.81	8.48	-12.66	0.119	
M_R_Omega	0.151	1.89	8.80	-8.35	0.080	
Omega	0.268	2.60	6.87	-12.87	0.103	
S&P 500	0.098	1.94	9.38%	-11.9	0.051	
Panel B: Market value						
Model	Final market value ($)	Average market value ($)	Average number of buying assets ($)	Average trading cost ($)	Accumulated trading costs ($)	Average turnover rate
M_Omega	2,578,948	1,949,123	8	643	167,888	0.150
R_Omega	2,178,821	1,640,495	8	573	149,563	0.156
M_R_Omega	1,974,260	1,699,791	16	506	264,114	0.199
Omega	1,847,485	1,684,863	8	160	42,368	0.006

**Table 6:** Portfolio comparisons for the NASDAQ 100 dataset from 1/1/2020 to 4/22/2021.

Panel A: Return						
Model	Average return (%)	Standard deviation (%)	Max return (%)	Min return (%)	Sharpe Ratio	
M_Omega	0.309	2.63	7.54	-12.58	0.118	
R_Omega	0.281	2.47	6.41	-10.12	0.114	
M_R_Omega	0.135	1.71	6.35	-8.45	0.079	
Omega	0.260	2.39	6.43	-10.84	0.109	
NASDAQ 100	0.159	2.12	10.07	-12.19	0.075	
Panel B: Market value						
Model	Final market value ($)	Average market value ($)	Average number of buying assets ($)	Average trading cost ($)	Accumulated trading costs ($)	Average turnover rate
M_Omega	2,109,424	1,735,226	7	249	67,306	0.071
R_Omega	1,982,629	1,662,045	7	243	65,507	0.070
M_R_Omega	1,909,386	1,632,104	14	184	99,433	0.080
Omega	1,862,297	1,620,649	7	114	30,725	0.043

**Table 7:** Portfolio comparisons for the S&P 500 dataset from 1/1//2018 to 4/22/2019.

Panel A: Return						
Model	Average return (%)	Standard deviation (%)	Max return (%)	Min return (%)	Sharpe Ratio	
M_Omega	0.063	1.66%	5.62%	-7.60%	0.038	
R_Omega	0.075	1.54%	8.33%	-6.07%	0.048	
M_R_Omega	0.029	1.09%	6.98%	-6.02%	0.026	
Omega	0.093	1.62%	6.81%	-9.42%	0.057	
S&P 500	0.031	1.02	4.96	-4.10	0.030	
Panel B: Market value						
Model	Final market value ($)	Average market value ($)	Average number of buying assets ($)	Average trading cost ($)	Accumulated trading costs ($)	Average turnover rate
M_Omega	1,131,902	1,197,801	11	342.78	87,409	0.125
R_Omega	1,174,102	1,199,331	11	340.20	86,750	0.123
M_R_Omega	1,122,951	1,184,847	22	307.25	156,699	0.179
Omega	1,220,162	1,247,058	11	66.27	16,898	0.032

**Table 8:** Portfolio comparisons for the NASDAQ 100 dataset from 1/1/2018 to 4/22/2019.

Panel A: Return						
Model	Average return (%)	Standard deviation (%)	Max return (%)	Min return (%)	Sharpe Ratio	
M_Omega	0.165	2.07%	9.80%	-11.07%	0.079	
R_Omega	0.156	1.98%	13.43%	-6.19%	0.079	
M_R_Omega	0.069	1.42%	10.95%	-7.36%	0.049	
Omega	0.159	2.25%	10.35%	-15.11%	0.071	
NASDAQ 100	0.061	1.35%	6.16%	-4.63%	0.045	
Panel B: Market value						
Model	Final market value ($)	Average market value ($)	Average number of buying assets ($)	Average trading cost ($)	Accumulated trading costs ($)	Average turnover rate
M_Omega	1,446,352	1,335,802	6	157	40,903	0.057
R_Omega	1,420,118	1,321,768	6	160	41,873	0.058
M_R_Omega	1,345,632	1,296,764	12	135	70,360	0.035
Omega	1,411,754	1,309,156	6	53	13,815	0.024

The most volatile duration (2020 to 2021) has standard deviations of 1.94 and 2.12 for the S&P 500 and NASDAQ 100 indexes, respectively. In this period, the Omega model combined with momentum (M_Omega) or reversal (R_Omega) performs best, though the M_Omega model outperforms the R_Omega model. Both the M_Omega model and the R_Omega model reach their highest final market value from 2020 to 2021, when the COVID 19 pandemic emerged. The huge growth in the final market value for the M_Omega model occurs with the S&P 500 dataset. This is consistent with the finding of Elaut et al. [15], that the effect of intraday momentum was prominent during financial crises.

Based on the final market value for the S&P 500 dataset, the M_Omega and R_Omega models show the greatest improvement over the Omega model, which are $731,463 and $331,336, respectively. Similarly, for the NASDAQ 100 dataset, the M_Omega and R_Omega improve over the Omega model by $247,127 and $120,332 respectively, as seen in Table 9.

**Table 9:** Differences in final market value for trading strategies relative to the Omega model.

Panel A: S&P 500			
Deviation	2018/1-2019/4	2020/1-2021/4	2021/1-2022/4
M_Omega to Omega	-$88,260	$731,463	-$11,457
R_Omega to Omega	-$46,060	$331,336	$47,332
Panel B: NASDAQ 100			
Deviation	2018/1-2019/4	2020/1-2021/4	2021/1-2022/4
M_Omega to Omega	$34,598	$247,127	$18,563
R_Omega to Omega	$8,364	$120,332	$25,594

Table 10 compares Sharpe ratios for the S&P 500 and NASDAQ 100 datasets. The Sharpe ratios of the M_Omega and R_Omega models outperform those of the Omega model and the S&P 500 index only during the period from 2020 to 2021. The Sharpe ratio of the R_Omega model is higher than that of the Omega model, but less than that of the S&P 500 index, from 2021 to 2022. This is due to the low standard deviation of the S&P 500 index return, 0.95, as the returns of the R_Omega and Omega models are higher than that of the S&P 500 index. In general, the NASDAQ 100 index is more volatile than the S&P 500 index based on standard deviation. However, comparing the Sharpe ratios for the NASDAQ 100 dataset, the M_Omega and R_Omega models outperform the Omega model and NASDAQ 100 index during three periods.

**Table 10:** Sharpe ratio comparisons.

Panel A: S&P 500			
Model	2018/1-2019/4	2020/1-2021/4	2021/1-2022/4
M_Omega	0.038	0.143	0.040
R_Omega	0.048	0.119	0.054
M_R_Omega	0.026	0.080	0.016
Omega	0.057	0.103	0.048
S&P 500	0.030	0.051	**0.060**
Panel B: NASDAQ 100			
Model	2018/1-2019/4	2020/1-2021/4	2021/1-2022/4
M_Omega	0.079	0.118	0.086
R_Omega	0.079	0.114	0.086
M_R_Omega	0.049	0.079	0.046
Omega	0.071	0.109	0.084
NASDAQ 100	0.045	0.075	0.018

Overall, the Omega model integrated with the momentum or reversal strategy is more profitable in volatile periods or markets than is the Omega model. The M_Omega and R_Omega models obtain their highest final market values in the most volatile period from 1/1/2020 to 4/22/2021 in both markets. Moreover, for the S&P 500 dataset, the Sharpe ratios of the M_Omega and R_Omega models outperform those of the Omega model and S&P 500 index only in the period from 2020 to 2021. For the NASDAQ 100 dataset, in contrast, the Sharpe ratios of the M_Omega and R_Omega models outperform those of the Omega model and NASDAQ 100 index for all three periods.

In addition, we also use the longer period 1/1/2019 to 6/27/2022 for additional testing. The experimental results of these three strategies over this longer period are not good because trading costs significantly erode market value. The momentum or reversal strategy thus seems suitable for short-term but not for long-term intraday trading. We also find that the previous intraday trading research often focuses on periods of less than one year. [32–35]. Also, high-tech stocks often become winners or losers because of the high fluctuations among these stocks. The proposed model overcomes the drawback that intraday trading frequently erodes profits, resulting in negative returns [25]. The M_R_Omega model suggests that performing the momentum and reversal strategies on the same day is not recommended because the frequent trading significantly erodes market value even though return reversal often follows return momentum [17].

## 4. Conclusions

This study integrates the momentum or reversal strategy into the Omega model, which incoporates gains and losses for intraday trading and daily rebalancing based on a 60-day historical return. Four findings emerge. First, the maximum number of invested assets for daily rebalancing should be limited to avoid the over-diversified problem. Second, the returns of M_Omega, R_Omega, and M_R_Omega are positive while limiting the number of invested assets, even considering the trading cost, in contrast with Herberger et al. [25]. The M_Omega and R_Omega models produce a higher return than that of the S&P 500 index or NASDAQ 100 index, considering the intraday trading cost. A shorter term trading strategy improves the results. Third, the Omega model integrated with the momentum or reversal strategy performs better in more volatile markets or periods. Finally, performing the momentum and reversal strategies on the same day is not recommended because frequent trading largely erodes the profit.

The data sets used in this work are the composite stocks of the S&P 500 and the NASDAQ 100. For further research, the proposed model can be extended to more data sets or other periods. Various momentum or reversal strategies with different ranking periods can be considered beyond the first half hour and first six half hours for the momentum and reversal strategies considered here. The proposed portfolio tends to be over-diversified if the number of invested assets is unlimited. Thus, finding the optimum number of invested assets in the intraday for the proposed Omega model will be worthwhile.
